# Impact of swimming pool water treatment system factors on the content of selected disinfection by-products

**DOI:** 10.1007/s10661-020-08683-7

**Published:** 2020-10-22

**Authors:** Joanna Wyczarska-Kokot, Anna Lempart-Rapacewicz, Mariusz Dudziak, Edyta Łaskawiec

**Affiliations:** Faculty of Energy and Environmental Engineering, Silesian University of Technology, Konarskiego 18 Street, Room 247, 44-100 Gliwice, Poland

**Keywords:** Trihalomethanes, Combined chlorine, Swimming pool water, Technological factors, Physicochemical factors

## Abstract

Recommendations regarding disinfection by-products (DBPs) in pool waters consider the content of trihalomethanes (THMs) and combined chlorine (CC) as indicators of DBPs based on which the health risk for swimmers and staff of pool facility can be determined. However, the content of DBPs in swimming pools depends on many factors. In this paper, the influence of selected factors (physicochemical parameters of water and technological parameters) on the content of THMs and CC in pool water was determined. During the 6-month period, 9 pools of various functions were analyzed. The water in pools was subjected to the same method of treatment. The content of THMs and CC was compared against the content of organic matter, free chlorine and nitrates, pH, temperature, redox potential and turbidity, technological, and operational parameters. The THM content did not exceed the limit value of 0.1 mg/L. The content of CC varied significantly, from 0.05 to 1.13 mg Cl_2_/L. It was found that a very large water volume per person, in comparison to a very small one, contributed to the low content of CC and THMs. The high load expressed as m^3^ of water per person or m^2^ of water per person and the specific function of hot tubs (HT1 and HT2) led to the average concentration of CC in these pools exceeding 0.3 mg Cl_2_/L. The THM concentrations in hot tubs (especially in HT1) were also among the largest (0.038–0.058 mg/L). In terms of the analyzed microbiological indicators, the quality of the tested pool water samples was not in doubt. It was found that the purpose of the pool, its volume, and number of swimmers should be the key parameters that determine the choice of methods of water treatment. The research on the pool water quality in the actual working conditions of swimming pool facilities is necessary due to the need to preserve the health safety of swimmers and staff.

## Introduction

Chlorination is the most commonly used disinfection approach for maintaining swimming pool water quality. Chlorine kills bacteria by attacking their lipids in the cell walls and destroying the enzymes and structures inside the cell. While the bacteria-killing properties of chlorine are very useful, they have some side effects. The chlorine solution poured into the water breaks down, among others, into hypochlorous acid (HOCl) and hypochlorite ion (OCl^−^). Once they are done killing the bacteria, they combine with another chemical. This process leads to the formation of disinfection by-products (DBPs) (Zwiener et al. 2007; Afifi and Blatchley 2015; Tardif et al. 2017) which pose a health risk for swimmers and additionally, due to volatility, also for lifeguards and staff of swimming pool facilities (Florentin et al. 2011; Fantuzzi et al. 2013; Abbasnia et al. 2018; Pándics et al. 2018). Chronic exposure to DBPs through different routes (inhalation, ingestion, and skin absorption) has been associated with an increased risk for bladder cancer (Jacobs et al. 2007; Kogevinas et al. 2010; Gallè et al. 2016). Several epidemiological studies have reported that health complications, associated with liver, reproductive system, kidney, and central nervous system, increased the risk of cancer due to consumption of water that contains DBPs; meanwhile, Dufour et al. indicated that an average swimmer ingests about 32 mL per hour of water while swimming, with a maximum range even up to 280 mL per hour (Dufour et al. 2017).

More than 600 different DBPs have been identified so far (Chowdhury et al. 2014; Manasfi et al. 2017; Tardif et al. 2017), including, for example, haloacids, halomethanes, haloacetonitriles, haloaldehydes, haloketones, halonitromethanes, haloamides, haloalcohols, halophenols, and also, non-halogenated contaminants, like benzaldehyde, benzeneacetonitrile, or phthalic acid. Among them, trihalomethanes (THMs), chloroform, and combined chlorine (CC) are widely referred to (Teo et al. 2015; Westerlund et al. 2015; Tardif et al. 2016). In swimming pools, combined chlorine (chloramines) is the most commonly identified. It is very troublesome for bathers, as it is responsible for the so-called “irritation syndrome” in swimmers, dry skin, and irritations of mucous membranes. It also causes the unpleasant odor of swimming pool water and has mutagenic properties (Villanueva et al. 2015; Gomà et al. 2017; Wyczarska-Kokot 2018). Only these three contaminants (combined chlorine, THMs, and chloroform as a part of THMs) are regulated by global and European documents in terms of swimming pool water quality (WHO 2006; DIN 19643–2012; DHM 2015). The most important swimming pool water quality requirements selected from these documents are summarized in Table 1.

**Table 1:** Regulations and guidelines of swimming pool water quality

Parameter	DHM 2015: Decree of the Health Minister on the requirements for water in swimming pools, JL 2015, item 2016 (Poland)	DIN 19643–2012: Treatment of water of swimming pools and baths - part 1: general requirements, German standard	WHO 2006: Guidelines for safe recreational water environments. Volume 2: swimming pools and similar environments
pH, −	6.5–7.6	6.5–7.2	7.2–7.8
Redox (mV)	> 750	> 750	> 720
Temperature (°C)	28–32	28–32	26–30
Turbidity (NTU)	0.3	0.5	0.5
Nitrates (mg NO_3_^−^/L)	20	20	-
COD^a^ (mg O_2_/L)	4	3	-
Free chlorine (mg Cl_2_/L)	0.3–0.6 (07–1.0)^b^	0.2–0.6 (07–1.0)^b^	< 1.2
Combined chlorine (mg Cl_2_/L)	0.3	0.2	0.2
THMs (mg/L)	0.1	0.02	0.1
*Escherichia coli* (CFU/100 mL)	0	0	0
*Pseudomonas aeruginosa* (CFU/100 mL)	0	0	0
*Legionella* sp. (CFU/100 mL)	0	0	0
Total number of bacteria, 36 ± 2 °C and 44 ± 4 h (CFU/1 mL)	100	100	200

^a^*COD*, chemical oxygen demand

^b^In pools equipped with hydro-massage devices, aerosol generating devices or in pools with a water temperature above 30 °C

Swimming pools are environments with high levels of DBPs due to continuous disinfection and constant organic load from bathers (Carter et al. 2019; Saleem et al. 2019; Tsamba et al. 2020). The presence and number of DBPs in swimming pools depends on many factors, including the method of water treatment, the type and concentration of used disinfectant, the content of natural organic matter, the physicochemical characteristics of treated waters, the contact time with bathers, attendance, and flow rates (Bradford 2014; Teo et al. 2015; Cheema et al. 2017; Tardif et al. 2017; Ilyas et al. 2018).

The main goal of this work was to determine the influence of selected factors (physicochemical parameters of water and technological parameters of processes related to the treatment system) on the content of THMs and combined chlorine in swimming pool water. The analyzed pools differ in terms of their intended use, the volume of the pool basin, usable area, filtration flow, and times of one water change in the pool basin. However, in all analyzed circuits, the water is subjected to the same method of treatment.

## Tested pools and water treatment systems

Thematic research was carried out for water samples taken from 9 swimming pools of various functions and technological parameters. The samples were taken from a sports pool (SP), a lesson swimming pool (LSP), an indoor recreational pool (IRP), an outdoor recreational pool (ORP), scuba-diving pool (SDP), a pupils’ pool (PP), laguna pool (with water attractions, LP), and two hydro-massage tubs (HT1 and HT2). The basic technical parameters for the tested pools are summarized in Table 2.

**Table 2:** Technical parameters of the tested swimming pools

Parameter	Type of the tested swimming pools								
	SP	LSP	IRP	ORP	LP	PP	SDP	HT1	HT2
Dimensions of the pool basin (m)	25 × 8.5	20 × 6	Irregular shape	11.5 × 10.0 irregular shape	11.5 × 10.0 irregular shape	11.5 × 9.0 irregular shape	Diameter 7.0	Diameter 3.0	3.0 × 2.1
Depth of the pool basin (m)	1.8	2.4	1.1–1.3	0.9–1.2	1.2	0.15–0.25	7.0	0.6	0.9
Volume of the pool basin (m^3^)	382.5	288.0	606.6	175.0	138.0	20.7	270.0	2.4	5.6
Usable area UA (m^2^)	212.5	120.0	527.5	137.0	110.4	103.5	38.0	4.2	6.3
Attendance (person/h)	16	10	19	18	17	4	4	4	6
Bather loads (m^2^/person)	13.3	12.0	27.8	7.6	6.5	25.9	9.5	1.1	1.1
Bather loads (m^3^/person)	23.9	28.8	31.9	9.7	8.1	5.2	67.5	0.6	0.9
Filtration flow (m^3^/h)	95	89	450	200	150	60	60	34	15
The time of one water change in the pool basin (h)	4.0	3.2	1.3	0.9	0.9	0.3	4.5	0.1	0.4
Water temperature (°C)	26	28	30	30	30	32	26	35	35

The tested pools are supplied with water from separate circulation systems, in which a continuous water treatment process takes place. The facility draws water from the municipal water supply system. The type of unit processes and equipment used in the water treatment circuits for individual pools is similar. The circuits are equipped with retention tanks and filtration systems.

**Fig. 1:**
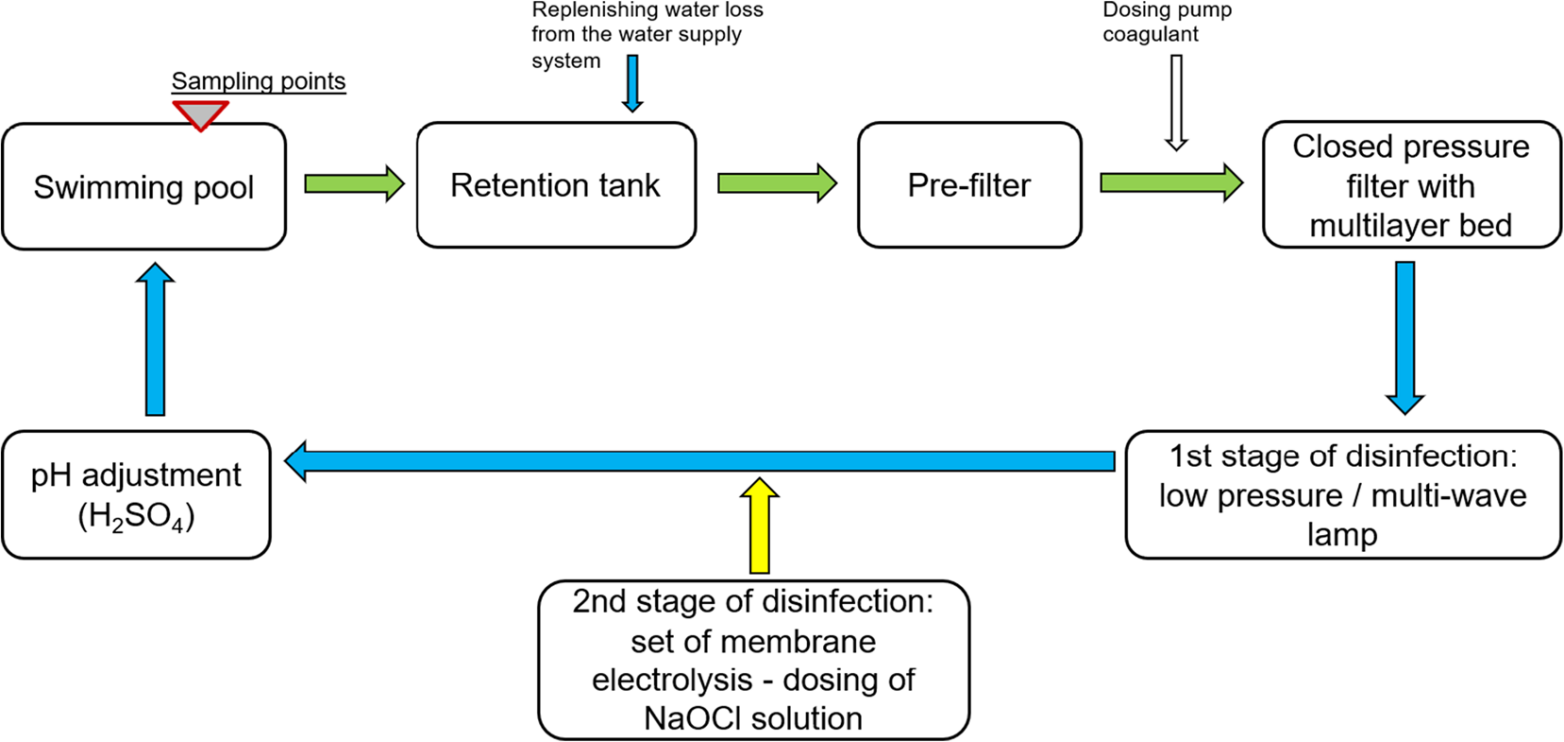
Water treatment system in tested swimming pools

The water from the retention tanks is collected using circulating pumps integrated with pre-filters, which stop larger impurities (fibers, hair, etc.), protecting pump impellers and other system components from damage. Water is pumped to closed pressure filters. The water is filtered with a speed of 30 m/h. The high efficiency of swimming pool water filtration is ensured by multi-layer beds consisting of several layers of different grain size and the total height of the bed from 1.2 to 1.5 m (DIN 2012). The analyzed pools have beds consisting of a layer of gravel, a layer of sand, and a layer of anthracite. The bed height in each filter is the same and it is 1.2 m. Washing of filtration beds is carried out with air (all filtration systems are equipped with air blowers) and water (taken from compensating tanks) and takes place at least every 3 days or when the pressure difference before and after the filters reaches 0.3 bar. Washing is carried out while there are no swimmers. In order to increase the filtration efficiency, a coagulant (0.5% aluminum hydroxychloride solution) was added before the filters. Based on the commissioning of the swimming pool water treatment plant and the expected load of bathers, the coagulant dose was determined in the range of 0.5–1.0 mL/m^3^. Water disinfection was carried out in two stages. First, the circulating water stream was subjected to physical disinfection by irradiation with UV rays (low pressure lamps for SP, LSP, IRP, ORP, LP, PP, SDP, and HT2 and multi-wave lamp for HT1, the intensity of irradiation: 600 J/m^2^), followed by chemical disinfection by dosing the sodium hypochlorite solution (produced in situ in the membrane electrolysis process). If the pH of the water needed adjustment, a 30% solution of sulfuric acid was used. The pool water treatment plants have been equipped with automatic systems for dosing the reagents (coagulant, NaOCl, H_2_SO_4_) and controlling basic water quality parameters (temperature, pH, redox, free chlorine, combined chlorine). The swimming pool basins are equipped with a vertical water flow system with an active overflow. Each of the pools is available for use for 16 h a day. The block diagram of water treatment for the tested pools is shown in Fig. 1.

## Materials and methods

Concentrations of trihalomethanes (THMs) and combined chlorine (CC) in combination with other parameters of pool water quality and factors influencing their value were analyzed in 9 pools (1 outdoor pool and 8 indoor pools) in Poland. During a 6-month period, basic and advanced analyses of the water quality were performed. The basic parameters were measured once a day and the advanced parameters once a week.

As part of the basic analyses, the values of water pH, temperature, and redox potential and the concentration of free chlorine and combined chlorine were measured. The advanced analyses included the concentration of nitrates, trihalomethanes (THMs), turbidity, chemical oxygen demand (COD), total organic carbon (TOC), absorbance UV_254_, CFU (colony forming units) of *Pseudomonas aeruginosa*, *Escherichia coli*, and *Legionella* sp., and a total number of mesophile microorganisms. Concentrations of THMs should be understood as the sum of the concentrations of trichloromethane (chloroform), bromodichloromethane, dibromochloromethane, and tribromomethane. The content of THMs and CC was compared against the content of organic matter (COD, TOC, and absorbance UV_254_), physicochemical parameters of water quality, and technological parameters of swimming pool (volume and usable area in relation to the number of swimmers, flow rate in relation to the time of one water change in the pool basin).

The main goal of the physical and chemical research was to determine the chemical composition of water, with special consideration of substances hazardous to health, such as trihalomethanes and combined chlorine. The main goal of the bacteriological research was an assessment of the risks from microorganisms that, regardless of the degree of contamination of the pool water by disinfection by-products (DBP), play a predominant role in the inspection process of swimming pool water quality. Comparison of the obtained results with the permissible levels of contamination for the pool water allowed to determine whether the water was suitable for bathing (WHO 2006; DIN 2012; DHM 2015).

The pH of water, redox, and temperature were measured by the potentiometric method in situ (sensION meter + MM150 DL, Hach®, Loveland, CO, USA). Concentrations of free, total, and combined chlorine were determined with a colorimetric method in situ (portable Pocket Colorimeter II DeviceTM, Hach®, Loveland, CO, USA). Concentrations of THMs were determined with the gas chromatography method (Agilent Technologies GC7890B chromatograph with MSD5977A mass detector, USA). Measurement of nitrates concentration and chemical oxygen demand value was made by using the photometric method in cuvette tests (DR 3900 spectrophotometer VIS with RFID technology, Hach®, Loveland, CO, USA). To determine the turbidity of samples, a nephelometric method was used (TN 100 turbidity meter, Eutech®, Singapore). Total organic carbon was measured using a TOC-L series analyzer by catalytic oxidation combustion at 680 °C (Shimadzu). The absorbance was measured at 254 nm, using a UV–visible light (UV–Vis) Cecil 1000 (Analytik Jena AG company, Jena, Germany), with an optical path length of the cuvette d equal to 1 cm. The absorbance UV_254_ value was determined based on the standards adopted by the US EPA–Method 415.3. (Potter and Wimsatt 2009). The total number of bacteria (in 36 ± 2 °C and 44 ± 4 h), *Pseudomonas aeruginosa*, *Escherichia coli*, and *Legionella* sp., was determined with the horizontal method using agar medium and membrane filtration, respectively (in accordance with unified methods listed in Table 3).

**Table 3:** Methodology of measuring physicochemical and bacteriological parameters

Parameter	Measurement based on the standard’s guidelines:	Accuracy of measurement
pH, −	PN-EN ISO 10523: 2012: water quality—determination of pH	± 0.01
Redox (mV)	PB-247/P ed. 3th of April 20, 2017 (measurement with an Ag/AgCl electrode in 3.5 M KCl); DIN 38404 part 6: determination of the oxidation reduction (redox) potential	± 1
Temperature (°C)	PB/BT/8/C:01.07.2018 (0.0–50.0) °C: water and sewage—temperature measurement	± 0.1
Turbidity (NTU)	PN-EN ISO 7027-1: 2016–09 point 5.3: water quality—determination of turbidity—part 1: quantitative methods	± 0.01
Nitrates (mg NO_3_^−^/L)	PN-EN ISO 13395: 2001: water quality—determination of nitrite and nitrate nitrogen and their sums by flow analysis (CFA and FIA) with spectrometric detection	± 0.0
COD (mg O_2_/L)	PN-EN ISO 8467: 2001: water quality—determination of chemical oxygen demand	± 0.5
Free chlorine (mg Cl_2_/L)	PN-EN ISO 7393-2: 2018–04: water quality—determination of free chlorine and total chlorine—part 2: colorimetric method with N, N-dialkyl-1,4-phenylenediamine, for routine control purposes	± 0.01
Combined chlorine (mg Cl_2_/L)	PN-EN ISO 7393-2: 2018–04: water quality—determination of free chlorine and total chlorine—part 2: colorimetric method with N, N-dialkyl-1,4-phenylenediamine, for routine control purposes. Indirect method (difference in total and free chlorine content)	± 0.01
THMs (mg/L)	PN-EN ISO 10301: 2002: water quality—determination of volatile halogenated hydrocarbons—methods using gas chromatography (HS-GCMS)	± 0.001
*Escherichia coli* (CFU/100 mL)	PN-EN ISO 9308-1: 2014–12: water quality—quantification of *Escherichia coli* and coliform bacteria—part 1: membrane filtration method for testing waters with a low amount of accompanying microflora	
*Pseudomonas aeruginosa* (CFU/100 mL)	PN-EN ISO 16266: 2009: water quality—detection and quantification of *Pseudomonas aeruginosa*—membrane filtration method	
*Legionella* sp. (CFU/100 mL)	PN-EN ISO 11731: 2017–08: water quality—quantification of *Legionella* bacteria	
Total number of bacteria, 36 ± 2 °C and 44 ± 4 h (CFU/1 mL)	PN-EN ISO 6222: 2004: water quality—quantification of microorganisms capable of growth—determination of the total number of bacteria by plating on nutrient agar	

### Quality control

Sampling took place in accordance with the guidelines of the unified standard PN-EN ISO 5667-3:2013-05 (Water quality - Sampling - Part 3: Preservation and handling of water samples). Samples were taken from a depth of about 30 cm below the water surface. The authors, guided by the experiments from previous studies, took samples at several characteristic basin points and used a mixed sample for analysis (Wyczarska-Kokot et al. 2020). The samples were collected and marked in accordance with applicable standards and methods (Down and Lehr 2005; Kaul 2007; Harvey et al. 2013; Schmitz 2016).

The analysis of the research results was based on the Microsoft Excel data analysis package.

Each sample was analyzed three times and the presented results are the average values of these repetitions. Standard deviations of repetitions did not exceed 5% that indicates a high repeatability of results.

The accuracy of the measurements and the mentioned parameters were assured by using the guidelines of the unified standards listed in Table 3.

The results of the analysis were compared with the guidelines included in the documents specifying the requirements for water in swimming pools (Table 1).

## Results and discussion

The THM content of the tested pool water samples did not exceed the limit value of 0.1 mg/L and ranged from 0.011 mg/L in PP to 0.058 mg/L in HT1. The content of combined chlorine varied significantly, from 0.05 mg Cl_2_/L in SDP to 1.13 mg Cl_2_/L in LP. In all pools, the permissible concentration was exceeded, i.e., 0.2 mg Cl_2_/L, according to the WHO guidelines and German standards (WHO 2006; DIN 2012). In turn, the exceedance of the permissible content of CC, i.e., 0.3 mg Cl_2_/L according to DHM 2015, was recorded in 6 out of 9 tested pools (SP, LSP, IRP, LP, HT1, and HT2).

An attempt was made to determine the variability of THMs and CC content in waters from pools of different intended use and depending on two types of indicators: firstly, depending on closely related indicators, technical (volume and usable area of the basin), technological (flow filtration), and operational ones (attendance) and secondly, depending on the physicochemical indicators of water quality.

### Technical, technological, and operational indicators

**Fig. 2:**
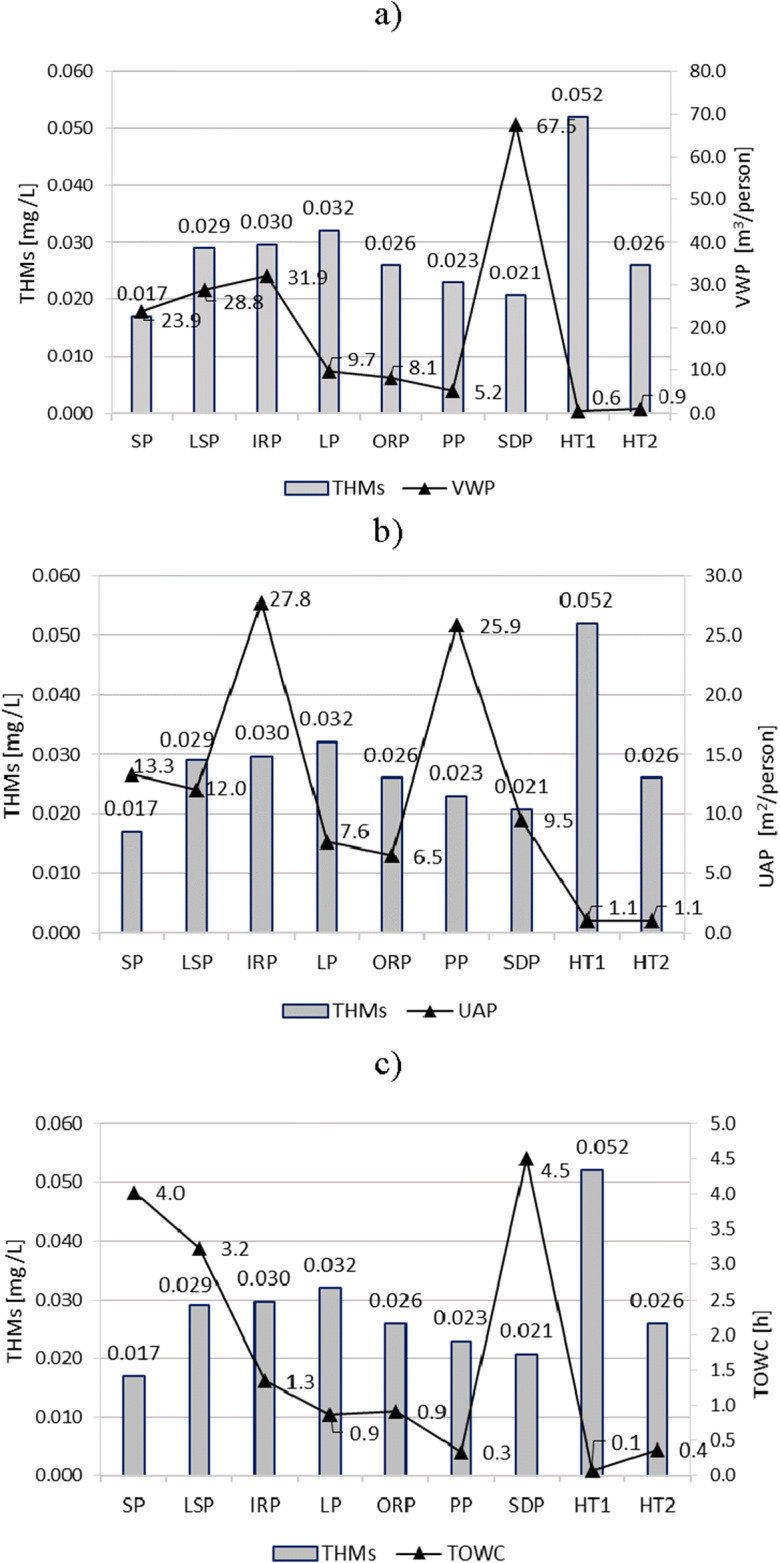
Dependence of the content of THMs on technological and operational parameters of swimming pools: **a** volume of water per person (VWP), **b** usable area per person (UAP), and **c** the time of one water change in the pool (TOWC)

**Fig. 3:**
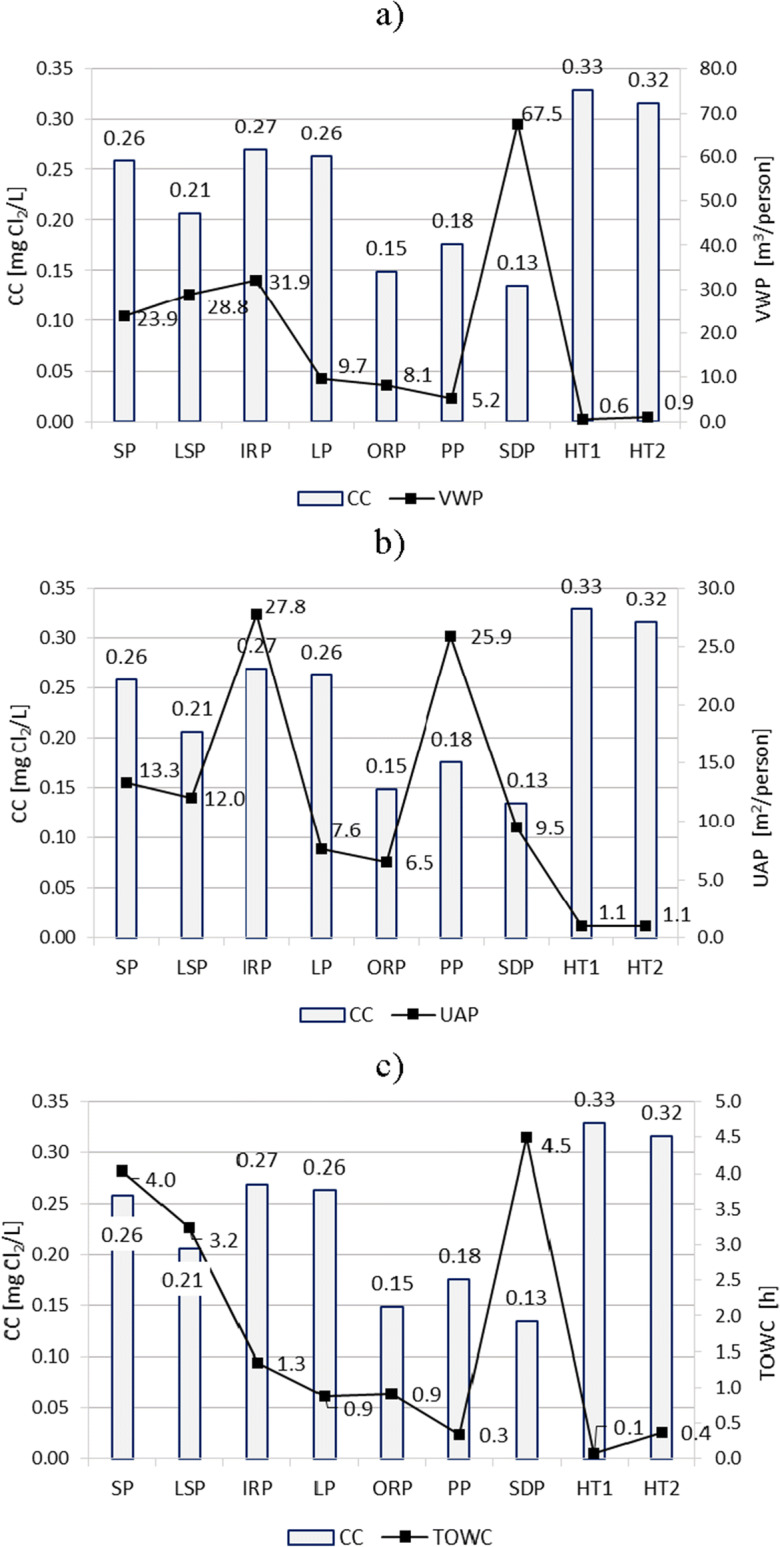
Dependence of the content of CC (combined chlorine) on technological and operational parameters of swimming pools: **a** volume of water per person (VWP), **b** usable area per person (UAP), **c** the time of one water change in the pool (TOWC)

The content of THMs and CC, depending on technical, technological, and operational indicators, has been determined in relation to the following:

- Bather loads (m^3^/person) pertaining to the volume of a swimming pool and average attendance, and described as volume of water per person (VWP) (Figs. 2a and 3a)
- Bather loads (m^2^/person) in relation to the usable area of water in the pool per person and average attendance, and described as a usable area per person (UAP) (Figs. 2b and 3b)
- The time of one water change in the pool basin (TOWC), that value resulting from filtration flow (m^3^/h) and volume of the pool basin (m^3^) (Figs. 2c and 3c)

As a result of the analysis, it was found that a very large VWP in the SDP pool (67.5 m^3^/person), in comparison to other pools, and especially to VWT in HT1 and HT2 pools (0.6 and 0.9 m^3^/person) contributed to the low content of CC (0.13 mgCl_2_/L) and THMs (0.021 mg/L). In this case, the low load of bathers, expressed indirectly as VWP in m^3^/person, correlated with low content of CC and THMs.

The high load expressed both as m^3^/person and m^2^/person and the specific function of HT1 and HT2 pools, despite the rapid change of water (0.1 h and 0.4 h), meant that in these pools, the average concentration of CC exceeded 0.3 mg Cl_2_/L. The THM concentrations in hot tubs (especially in HT1) were also among the largest (0.038–0.058 mg/L).

In the case of typical recreational swimming pools (equipped with hydro-massage devices, fountains, and water slides—IRP, LP, ORP, and PP), it was possible to state that a short time of full water change in the pool, with relatively heavy bather load (especially in LP and ORP), resulted in a low level of CC and THMs.

Despite the significant difference in the bather load in SDP (67.5 m^3^/person) and ORP (8.1 m^3^/person), both contained a low level of CC (0.13 and 0.15 mg Cl_2_/L on average). The susceptibility of chloramines (especially trichloramine) to be released from water into the air is undoubtedly considered to be the cause of such a state (Yue et al. 2016).

The presented case study is a new approach to assessing the quality of swimming pool water which, although supported by research in the field of swimming pool water system modeling and swimming pool water treatment (Orlov et al. 2018; Golbaz et al. 2019; Saleem et al. 2019), indicates that the guidelines for the design of swimming pools should be reviewed.

### Physicochemical indicators

The content of THMs and CC depending on physicochemical indicators of water quality has been determined with pH, redox potential, temperature, turbidity, nitrates, absorbance, COD, TOC, and free chlorine.

Values of mean, standard deviations (SD), and the range of obtained values of physicochemical parameters determining swimming pool water quality are presented in Tables 4 and 5.

**Table 4:** Physicochemical indicators of water contamination in tested swimming pools (SP, LSP, IRP, and LP)

Pool	SP		LSP		IRP		LP	
Parameter	Mean ± SD	Range	Mean ± SD	Range	Mean ± SD	Range	Mean ± SD	Range
pH, −	7.2 ± 0.1	7.1–7.3	7.2 ± 0.1	7.1–7.3	7.2 ± 0.1	7.1–7.3	7.1 ± 0.1	7.0–7.3
Redox (mV)	779 ± 61	719–900	761 ± 22	734–789	772 ± 211	745–803	775 ± 15	757–794
Temperature (°C)	29.0 ± 0.2	28.6–29.1	30.5 ± 0.3	30.2–31.0	30.1 ± 0.4	29.7–31.0	32.3 ± 0.5	31.7–33.1
Turbidity (NTU)	0.25 ± 0.00	0.25–0.26	0.32 ± 0.05	0.20–0.34	0.22 ± 0.03	0.20–0.28	0.23 ± 0.01	0.20–0.24
Nitrates (mg NO_3_^−^/L)	7.4 ± 0.3	6.6–7.5	7.06 ± 0.6	6.4–8.2	22.3 ± 2.1	21.3–27.0	10.0 ± 1.5	9.3–13.3
Absorbance UV_254_ (m^−1^)	0.023 ± 0.004	0.020–0.028	0.029 ± 0.001	0.028–0.031	0.025 ± 0.003	0.020–0.028	0.0180 ± 0.001	0.017–0.019
COD (mg O_2_/L)	1.9 ± 0.4	1.2–2.4	2.3 ± 0.3	1.9–2.5	2.3 ± 0.6	1.2–3.0	2.7 ± 0.7	1.2–3.5
TOC (mg C/L)	3.235 ± 0.004	3.230–3.240	7.441 ± 0.120	7.355–7.611	3.164 ± 0.039	3.115–3.210	3.739 ± 0.075	3.680–3.845
Free chlorine (mg Cl_2_/L)	0.41 ± 0.11	0.28–0.60	0.42 ± 0.11	0.28–0.58	0.50 ± 0.08	0.40–0.64	0.79 ± 0.09	0.68–0.94
Combined chlorine (mg Cl_2_/L)	0.26 ± 0.06	0.15–0.36	0.21 ± 0.15	0.06–0.80	0.27 ± 0.05	0.15–0.36	0.26 ± 0.21	0.09–1.13
THMs (mg/L)	0.017 ± 0.002	0.013–0.019	0.029 ± 0.005	0.022–0.034	0.030 ± 0.005	0.021–0.034	0.032 ± 0.001	0.031–0.034

**Table 5:** Physicochemical indicators of water contamination in tested swimming pools (ORP, PP, SDP, HT1, and HT2)

Pool	ORP		PP		SDP		HT1		HT2	
Parameter	Mean ± SD	Range	Mean ± SD	Range	Mean ± SD	Range	Mean ± SD	Range	Mean ± SD	Range
pH, −	7.2 ± 0.0	7.1–7.2	7.1 ± 0.2	6.6–7.3	7.2 ± 0.1	7.0–7.3	7.1 ± 0.2	6.8–7.3	7.2 ± 0.1	7.0–7.3
Redox (mV)	785 ± 8	771–799	743 ± 24	718–792	781 ± 6	772–790	788 ± 10	774–802	783 ± 27	737–818
Temperature (°C)	30.5 ± 0.8	29.8–32.1	32.6 ± 0.3	32.1–33.1	30.5 ± 1.2	29.0–32.1	35.3 ± 0.4	34.7–35.9	35.7 ± 0.7	35.0–36.9
Turbidity (NTU)	0.32 ± 0.05	0.20–0.34	0.22 ± 0.01	0.22–0.24	0.21 ± 0.04	0.15–0.028	0.39 ± 0.24	0.12–0.81	0.52 ± 0.18	0.26–0.81
Nitrates (mg NO_3_^−^/L)	22.7 ± 3.1	21.3–29.7	10.2 ± 2.9	8.9–16.8	3.9 ± 0.1	3.8–4.1	6.4 ± 0.3	6.2–7.1	4.1 ± 1.3	2.5–6.1
Absorbance UV_254_ (m^−1^)	0.030 ± 0.002	0.028–0.032	0.020 ± 0.003	0.016–0.024	0.007 ± 0.001	0.006–0.008	0.110 ± 0.050	0.065–0.180	0.086 ± 0.009	0.075–0.098
COD (mg O_2_/L)	2.1 ± 0.5	1.1–2.6	2.9 ± 0.5	2.2–3.8	1.1 ± 0.1	1.0–1.4	3.3 ± 0.3	3.1–3.9	3.4 ± 0.5	2.7–4.2
TOC (mg C/L)	2.525 ± 0.061	2.451–2.600	7.474 ± 0.080	7.361–7.540	0.993 ± 0.067	0.898–1.048	7.008 ± 0.205	6.720–7.178	6.966 ± 0.060	6.881–7.018
Free chlorine (mg Cl_2_/L)	0.63 ± 0.18	0.34–1.00	0.41 ± 0.11	0.26–0.58	0.45 ± 0.09	0.28–0.59	0.82 ± 0.20	0.31–1.02	0.90 ± 0.15	0.69–1.21
Combined chlorine (mg Cl_2_/L)	0.15 ± 0.03	0.10–0.21	0.18 ± 0.05	0.09–0.27	0.13 ± 0.05	0.05–0.27	0.33 ± 0.04	0.23–0.40	0.32 ± 0.04	0.22–0.38
THMs (mg/L)	0.026 ± 0.008	0.014–0.032	0.023 ± 0.007	0.011–0.029	0.021 ± 0.001	0.019–0.021	0.052 ± 0.003	0.038–0.058	0.026 ± 0.002	0.021–0.027

### pH

It is very important to closely monitor and control pool water pH to ensure disinfection effectiveness and the smallest potential for forming THMs and chloramines. In the majority of tested pools, the water pH, thanks to precise automatic control and dosing of sulfuric acid solution for pH correction, was in a very narrow range, from 7.0 to 7.3. The largest pH range occurred in the PP and HT1 (pH = 6.6–7.3).

**Fig. 4:**
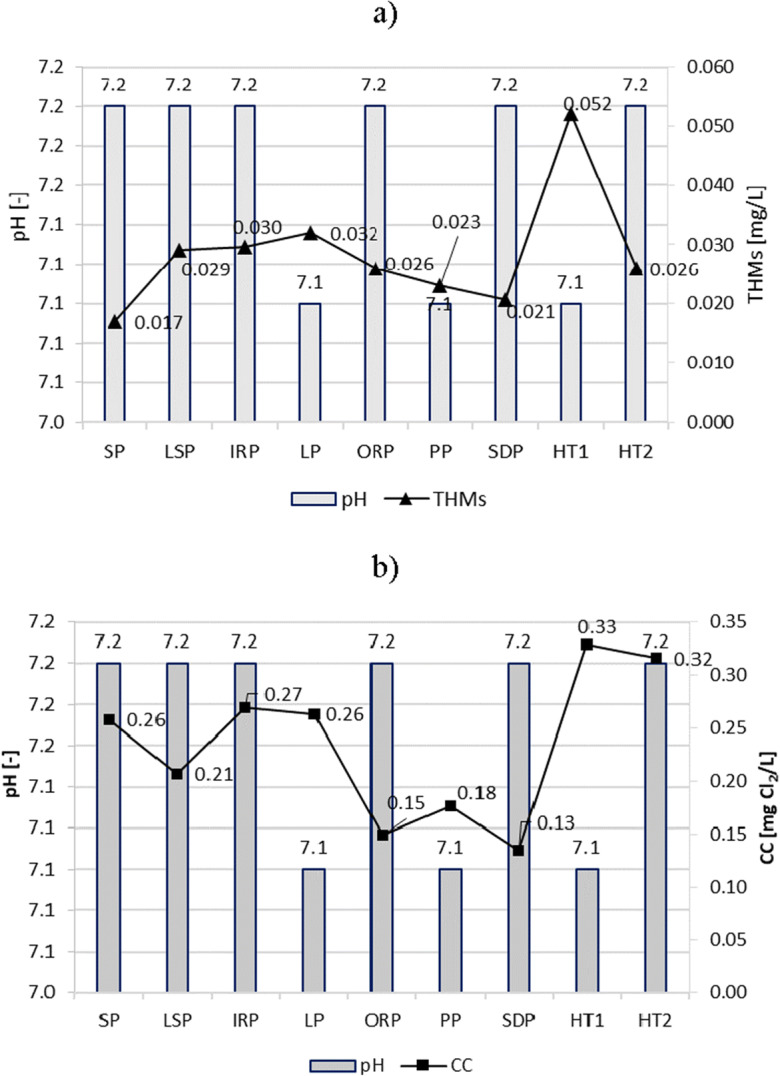
pH impact on content of **a** THMs and **b** CC in pool water

As in the research by Hansen et al. and Schmalz et al., the DBPs content was shown to depend on the pH value of swimming pool water (Schmalz et al. 2011; Hansen et al. 2012). The tested water samples showed a correlation between pH and THMs at the level *r* = − 0.55 (*r*—correlation coefficient). In the case of combined chlorine, this dependence was at a much lower level *r* = − 0.24. At both pH = 7.1 and pH = 7.2, small and high levels of CC (e.g., 0.33 mg Cl_2_/L in HT1 at pH = 7.1, 0.15 mg Cl_2_/L in ORP at pH = 7.2) and THMs (e.g., 0.052 mg/L in HT1 at pH = 7.1; 0.017 mg/L in SP at pH = 7.2) were determined (Fig. 4a, b).

### Redox potential

**Fig. 5:**
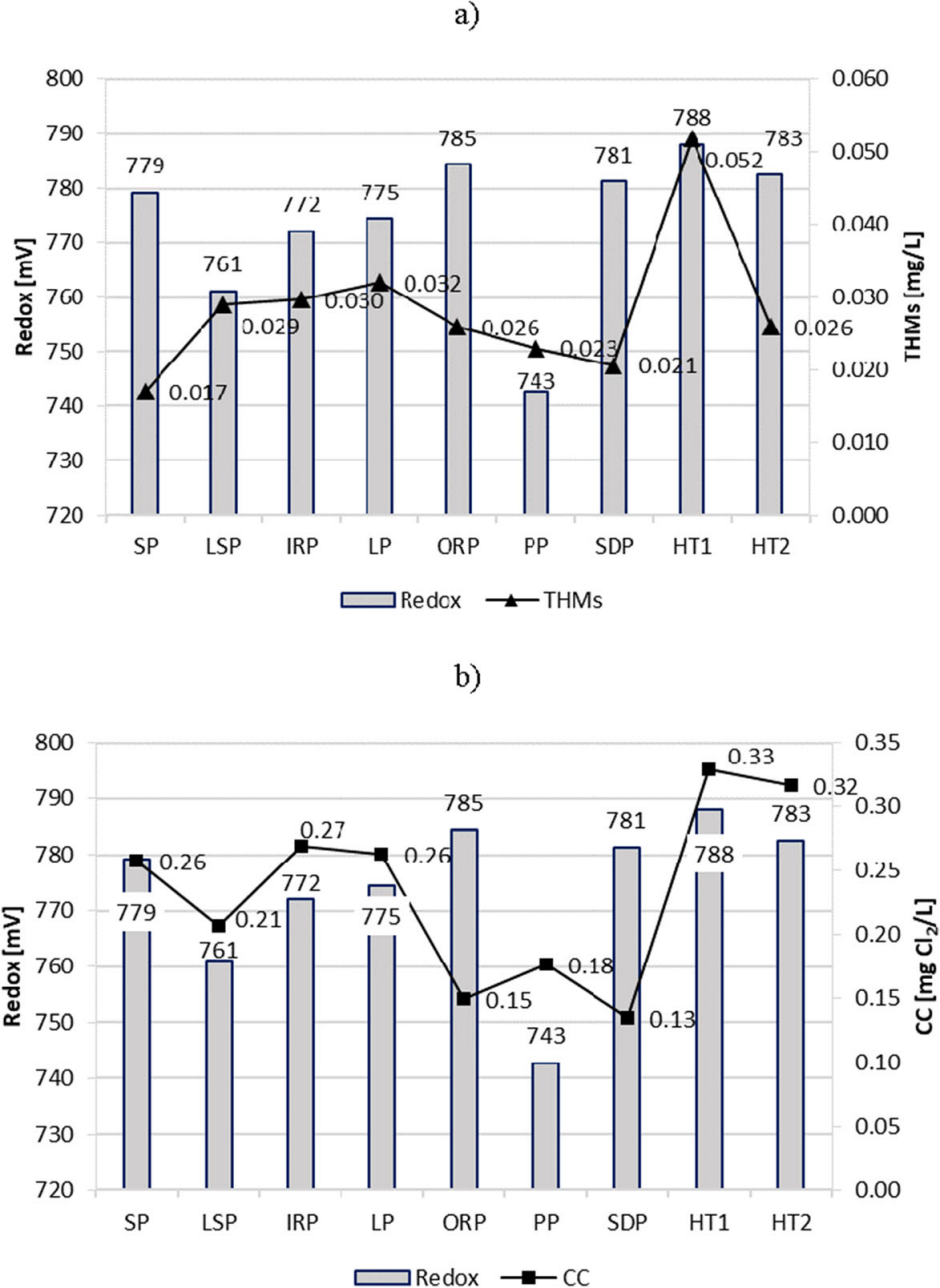
Redox potential impact on content of **a** THMs and **b** CC in pool water

The redox potential serves as an indication of the conditions in a water environment in connection with which oxidizing and reducing substances become active. The recommended value of redox potential in swimming pool water should not be less than 750 mV (DIN 2012; DHM 2015). In the tested samples, the redox potential ranged from 718 mV in PP to 900 mV in SP. The average redox values in all pools corresponded to the guidelines. However, it was difficult to state unequivocally to what extent the high or low potential influenced the reduction or increase of THMs and CC content. At the high redox value in the ORP, SDP, HT1, and HT2 (> 780 mV), significantly different CC contents were measured. In ORP and SDP, 0.15 and 0.13 mgCl_2_/L, in HT1 and HT2, 0.33 and 0.32 mgCl_2_/L. Also, in the case of THMs with relatively high redox, significant differences in their content were noted (e.g., in HT1 and SP) (Fig. 5a, b).

### Temperature

According to numerous studies (Keuten et al. 2012; Tang et al. 2013; Carter and Joll 2017), a higher pool temperature affects the faster release of human input, increased volatilization rate of volatile DBPs, and formation reactions of DBPs. Also, for pools operating at elevated temperatures, a higher content of disinfectants (0.7–1.0 mg Cl_2_/L) is required, which can also affect the formation of DBPs.

**Fig. 6:**
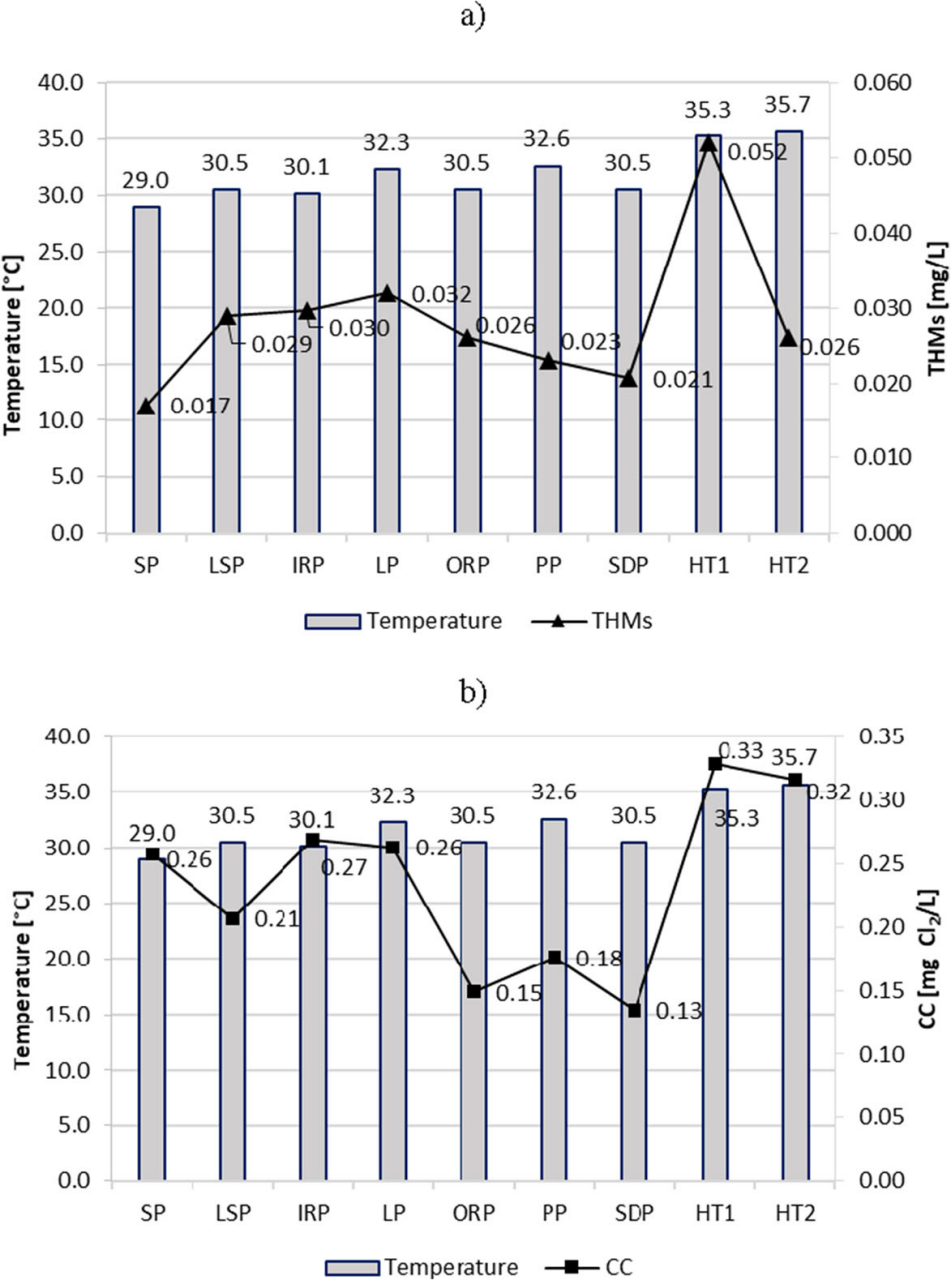
Temperature impact on content of **a** THMs and **b** CC in pool water

In the present studies, it was also possible to notice the effect of the increased water temperature on the increased content of CC and THMs. In HT1 and HT2 pools with a water temperature > 35 °C, despite high free chlorine concentrations (0.82 and 0.90 mg Cl_2_/L), the average content of CC was the highest of those measured and > 0.3 mg Cl_2_/L. The same was true for THM content in HT1 (Fig. 6a, b).

### Turbidity, absorbance UV_254_, COD, and TOC

The turbidity, absorbance UV_254_, COD, and TOC are important and related parameters that indicate the concentration of organic substances, especially those compounds that contain aromatic rings (Nowacka and Włodarczyk-Makuła 2016). These compounds are known as the main precursors for the formation of DBPs, including THMs and chloramines (Kanan and Karanfil 2011; Bradford 2014).

**Fig. 7:**
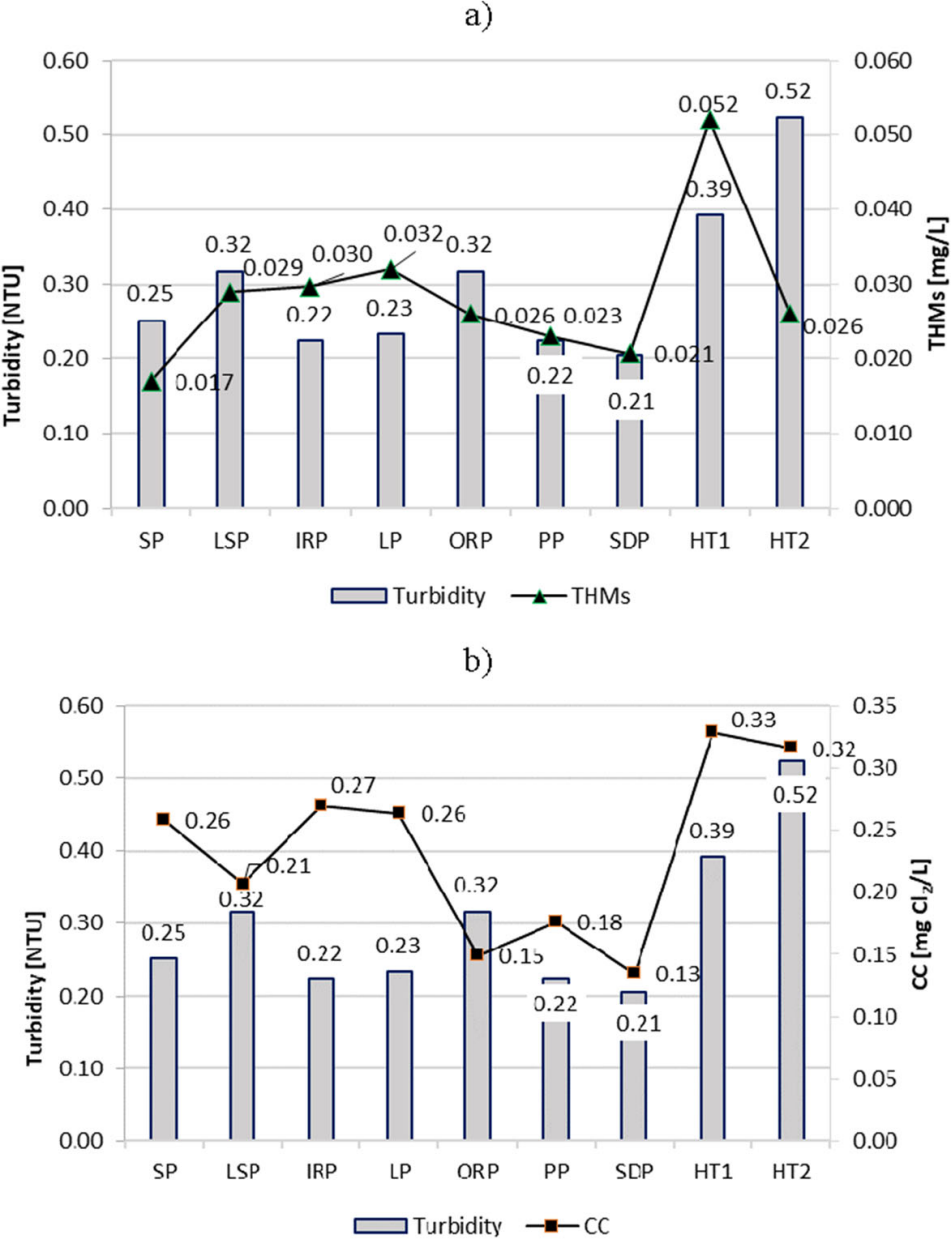
Turbidity impact on content of **a** THMs and **b** CC in pool water

**Fig. 8:**
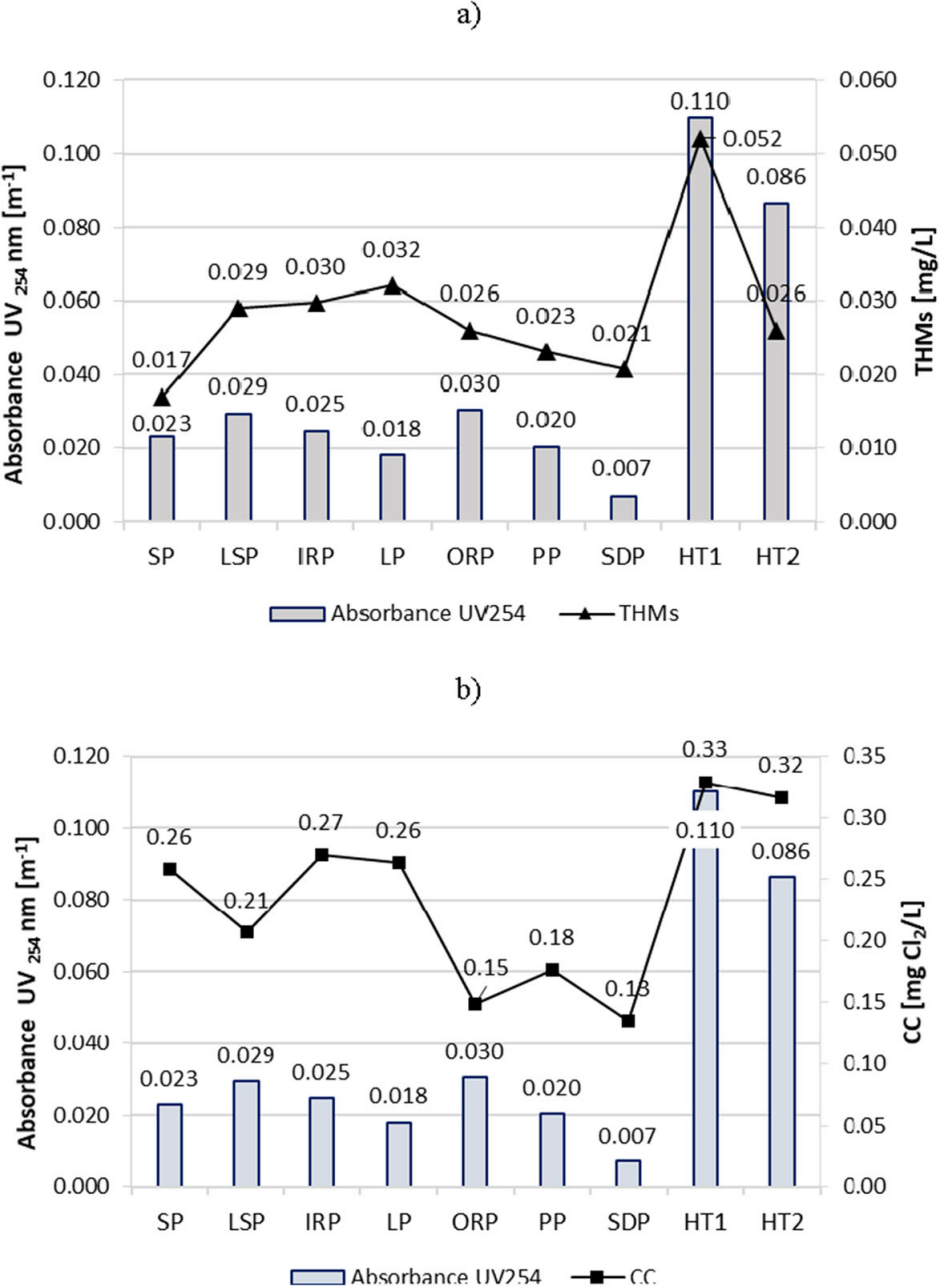
Absorbance UV_254_ impact on content of **a** THMs and **b** CC in pool water

**Fig. 9:**
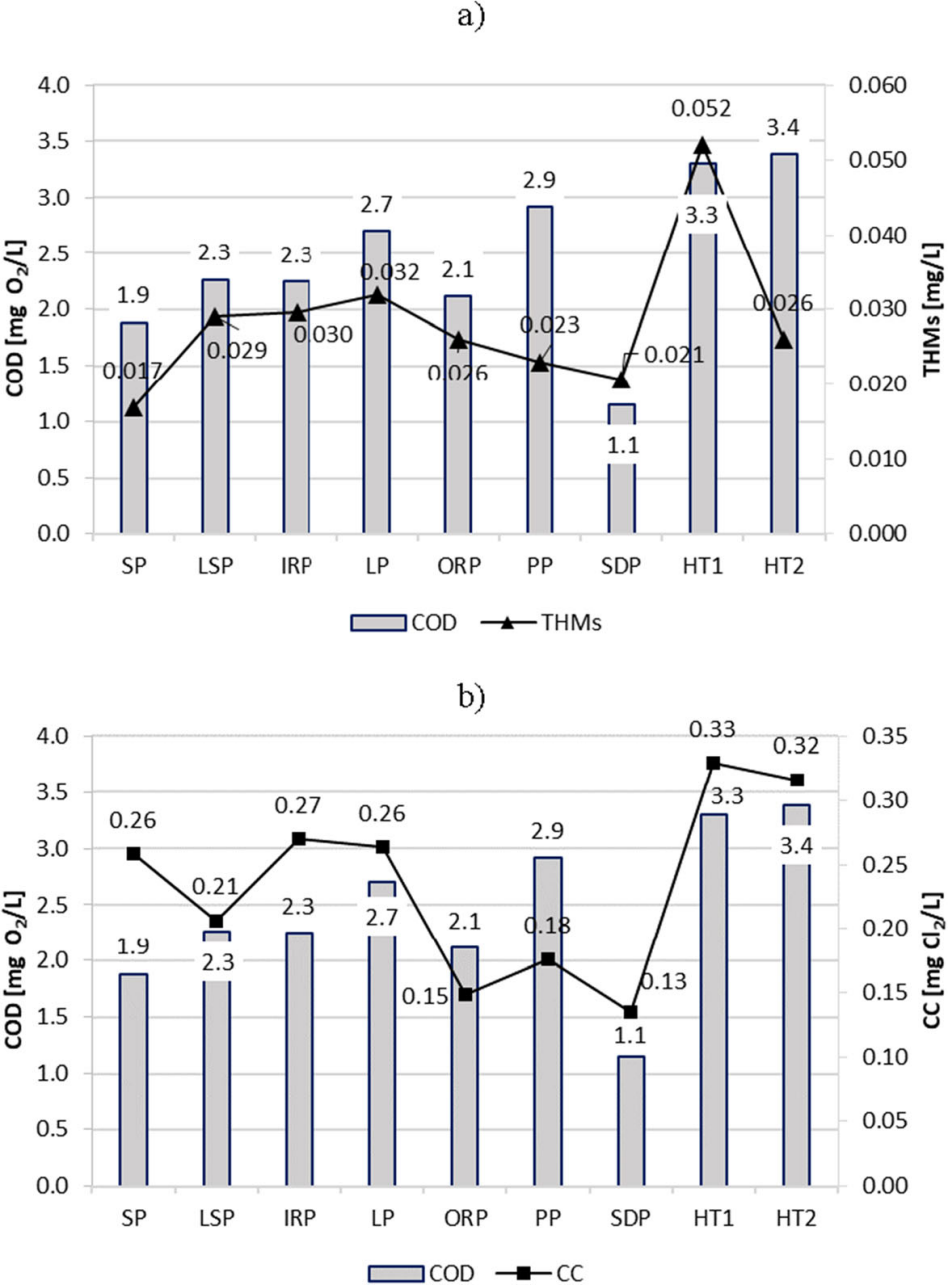
COD (chemical oxygen demand) impact on content of **a** THMs and **b** CC in pool water

**Fig. 10:**
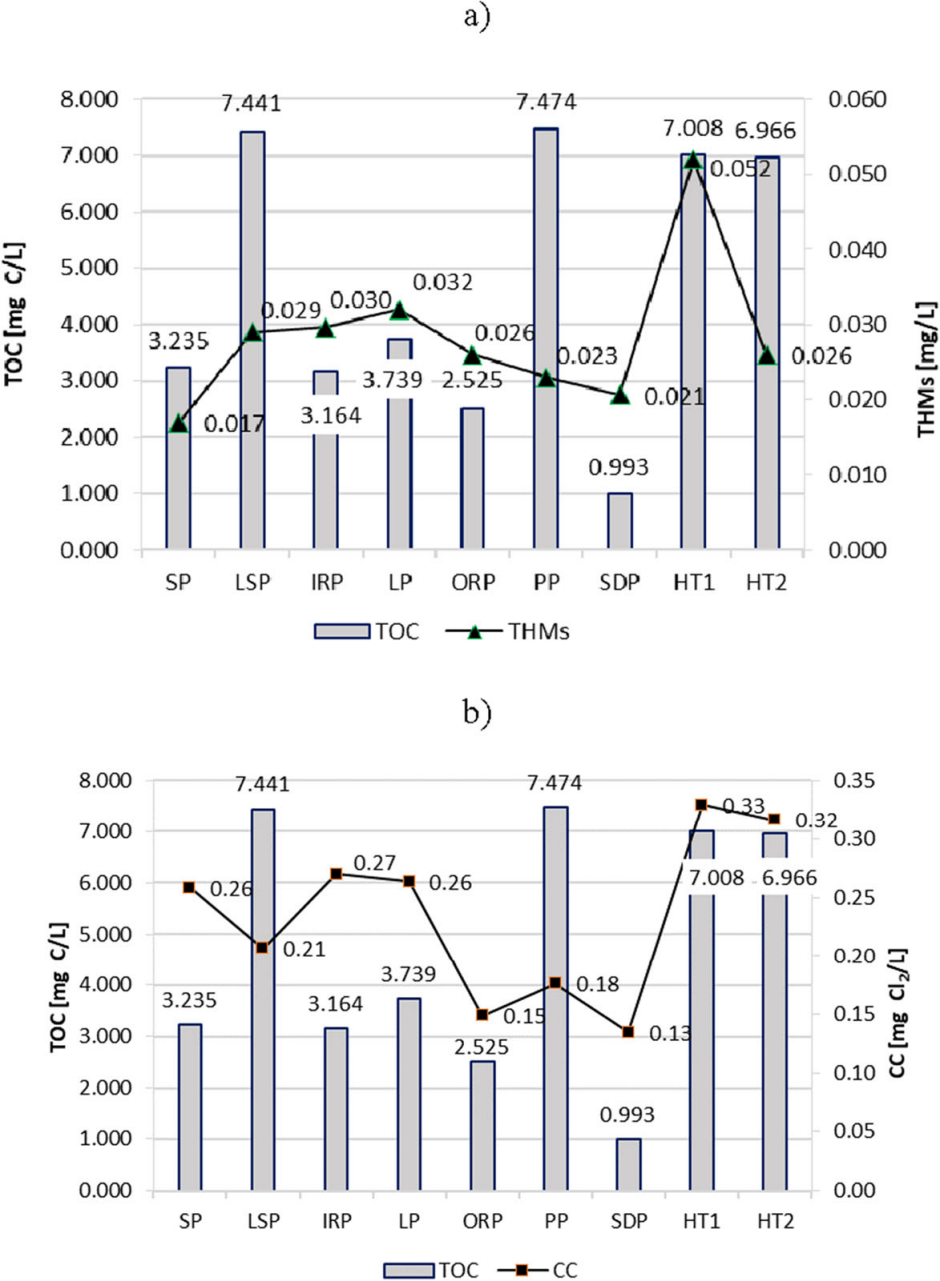
TOC (total organic carbon) impact on content of **a** THMs and **b** CC in pool water

The turbidity (Fig. 7), absorbance UV_254_ (Fig. 8), COD (Fig. 9), and TOC (Fig. 10) parameters showed similar trends in all tested swimming pools. However, the most visible relationship between these parameters was observed in the pools in which they reached the highest values, i.e., in HT1 and HT2. As a result, the THMs and CC contents of these pools were also the largest.

The largest variation was observed in the TOC index. As shown in the Judd and Bullock studies on swimming pools, organic pollutants could react with chlorine over a long time as the residence time of the pool water (the water exchange) lasted from weeks to months. As the TOC level in swimming pools does not increase significantly over time, it can be assumed that organic material added to the pool water by bathers reacts with chlorine to form DBPs (Judd and Bullock 2003).

In view of the current state of knowledge and conclusions from many studies in the field of improving the quality of pool water, the methods allowing for the reduction of the level of its organic contamination and thus the potential to create DBPs are the inclusion of membrane filtration (ultrafiltration, microfiltration or nanofiltration) and alternative processes of disinfection (electrochemically generated mixed oxidants, ultraviolet irradiation, and UV-based advanced oxidation processes, such as UV/H_2_O_2_, ozone, and ozone-based advanced oxidation processes, such as O_3_/H_2_O_2_ and O_3_/UV) in pool water treatment systems (Barbot and Moulin 2008; Cheema et al. 2017; Ekowati et al. 2019).

### Nitrates

It is well known that disinfection of pool water causes the formation of by-products as a result of chemical reactions between chlorine and nitrogen compounds that are part of the organic matter from swimmers. Indicators of pool water contamination with nitrogen compounds are in particular nitrates, as products of oxidation of ammonia—component of urine and sweat (De Laat et al. 2011; Bradford 2014; Teo et al. 2015).

**Fig. 11:**
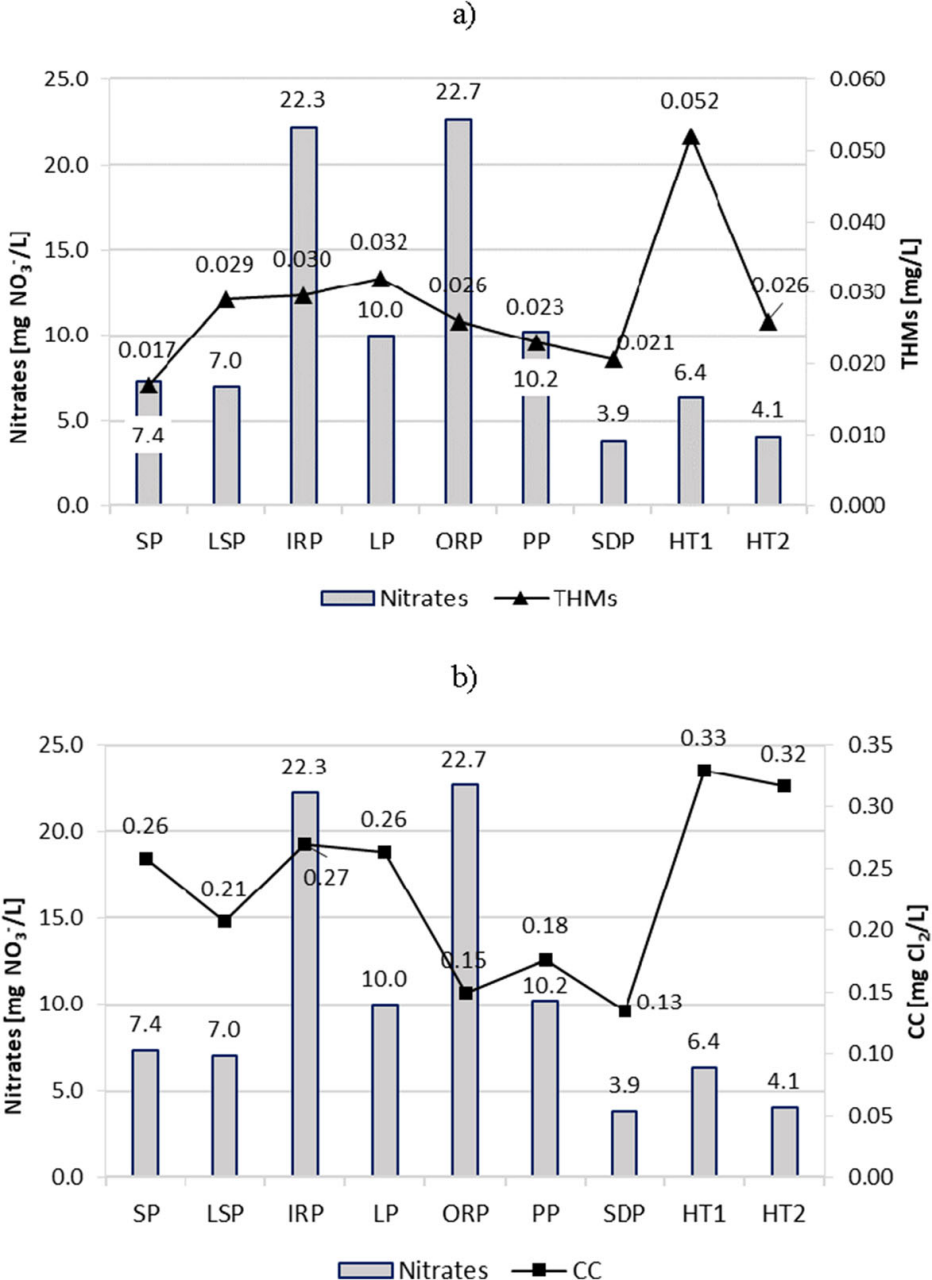
Nitrates impact on content of **a** THMs and **b** CC in pool water

The average concentration of nitrates found in tested pools was from 3.9 to 22.7 mg/L (Fig. 11).

Low content of nitrates in SDP (3.9 mg/L), in HT2 (4.1 mg/L), and in HT1 (6.4 mg/L) resulted most probably from fast water exchange in HT1 and HT2 and very low bather loads in SDP. In turn, very high content of nitrates in IRP (22.3 mg/L) and ORP (22.7 mg/L) was caused by high attendance, and in the case of ORP an additional load of pollutants from the vicinity of the pool beach. A comparative analysis of the content of THMs and CC on the nitrate content did not show a significant correlation between them. For example, high concentrations of combined chlorine were observed in water from HT1 and HT2 with low content of nitrates. In turn, low CC levels were observed in water from ORP with a high content of nitrates, while in the IRP having a high content of nitrates, low CC contents were observed.

### Free chlorine

The content of free chlorine in swimming pool water should amount to 0.3–0.6 mg Cl_2_/L. In the pools equipped with a blower and water jets, the concentration of free chlorine should be 0.7–1.0 mg Cl_2_/L, and in pools for children under 3 years old 0.3–0.4 mg Cl_2_/L (WHO 2006; DIN 2012; DHM 2015).

**Fig. 12:**
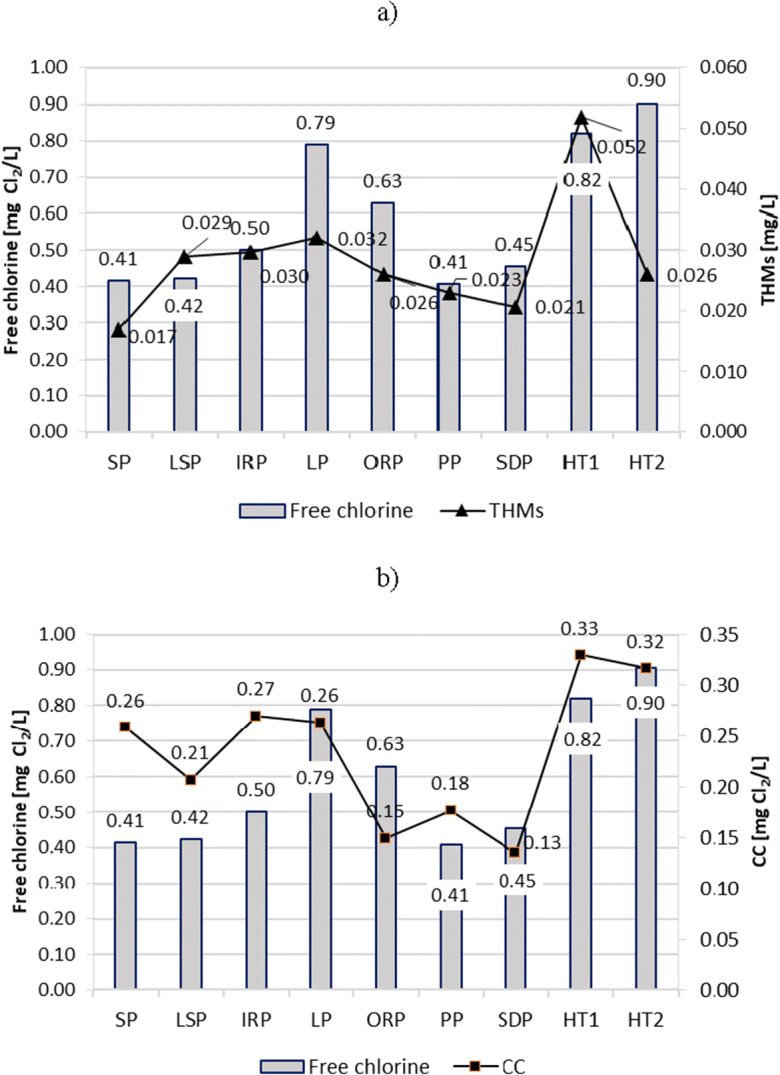
Free chlorine impact on content of **a** THMs and **b** CC in pool water

Free chlorine was regularly and automatically analyzed online and, at least once a day, using a photometer. In the event of a significant variation in the measurement results, it was possible to calibrate the measuring devices and adjust the dose of the NaOCl solution to the assumed values. Despite maintaining constant concentrations of free chlorine in individual pools, no significant differences were observed in the CC or THMs decrease in the different ranges of free chlorine concentrations (Fig. 12).

It should be emphasized, however, that with varying operating conditions of basins over a 24-h period, the control and measurement devices allowed for maintaining constant free chlorine concentration that guaranteed the adequate sanitizing conditions of the pool water.

### Microbial analysis

Due to the possibility of rapid spreading of diseases in the pool water environment, the decisive role in the assessment of its quality is played by the results of microbiological tests and the evaluation of the sanitary state of the facility (Masoud et al. 2016; Aboulfotoh Hashish et al. 2017; Wei et al. 2018).

**Fig. 13:**
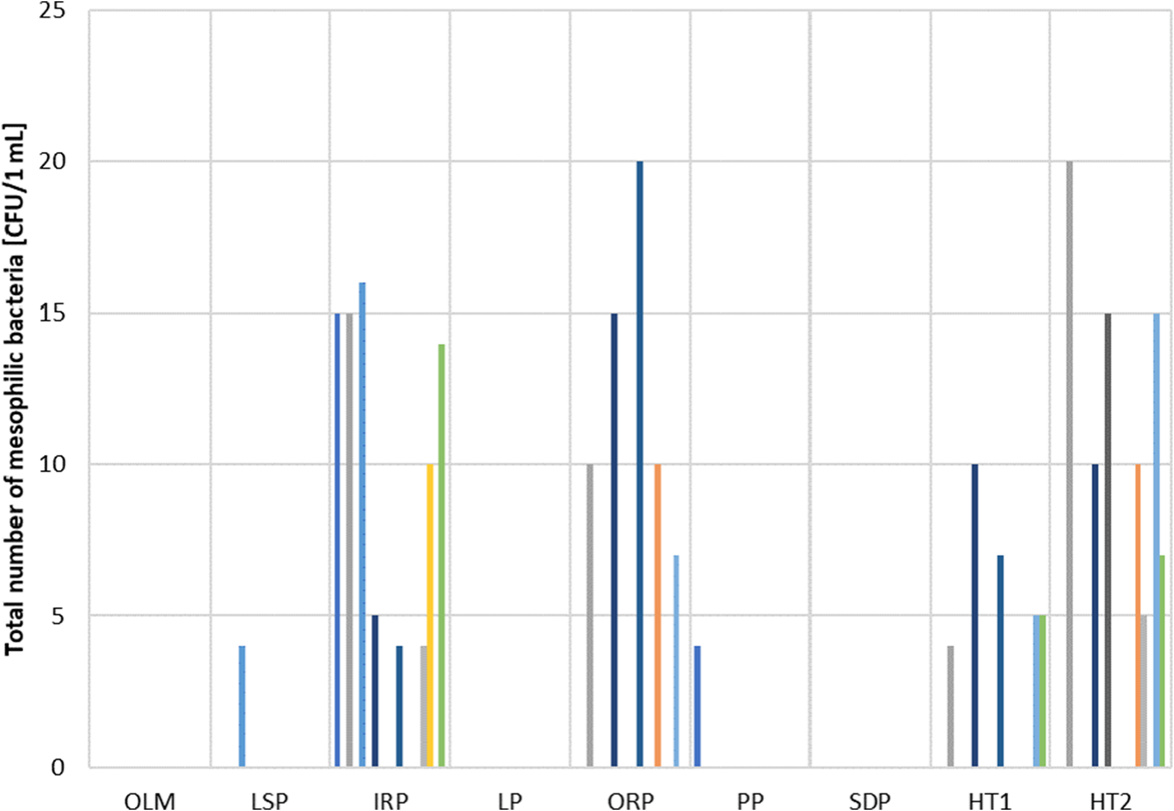
Total number of mesophilic bacteria in water samples from tested pools

Microbiological analyses performed for the considered pools did not show the presence of CFU of bacteria above the limits specified in the relevant regulations. No *Escherichia coli*, *Pseudomonas aeruginosa*, and *Legionella* sp. were found in the entire test cycle. The total number of mesophilic bacteria in water samples from the tested pools did not exceed the limit value of 100 CFU/1 mL. In IRP samples, a maximum of 16 CFU/1 mL was determined, in samples from ORP a maximum of 20 CFU/1 mL, and in samples from HT1 10 CFU/1 mL and from HT2 20 CFU/1 mL. In samples from other pools, the total number of mesophilic bacteria did not exceed 4 CFU/1 mL (Fig. 13).

## Conclusions

The function of a given swimming pool, its volume, and number of swimmers should be key parameters determining the selection of a water treatment method. In public swimming pools, it is required that the water is treated with chlorine compounds. This treatment should provide a quick and long-lasting bacteriological effect with the least possible potential for the creation of toxic DBPs, i.e., trihalomethanes and combined chlorine. On the basis of the results, it was determined that the levels of CC and THMs were higher in tubs with hydro-massage, the pupils’ pool, and indoor recreational pool, primarily due to a small volume of these pools in relation to the number of swimmers (daily bather loads). These pools showed also a higher content of TOC and COD indicator. In the case of swimming pools with devices for hydro-massage and other water attractions, the effectiveness of the treatment process should also be increased, e.g., by incorporating the processes of ultrafiltration, ozonization, and/or other hybrid processes.

The content of THMs and combined chlorine in pool waters is varied and depends on many factors. Both other pool water quality parameters, as well as technical and technological parameters, have an impact on their concentrations in the pool water. The analysis of the content of disinfection by-products (with particular reference to representative products such as THMs and CC) depending on the swimming pool’s operating conditions is important for the control of swimming pool water quality in terms of its health safety for swimmers. Such analysis also allows to control the reliability of running swimming pool water treatment processes, giving information on the accumulation of DBPs in a closed water pool circuit. In the field of pool water treatment technology, tests are required that take into account the specificity of operation of a given type of swimming pool facility with regard to the degree of water pollution acceptable for it. It should take into consideration both the design aspects of pool basins and pools halls (including the water circulation system in the basin, ventilation, and air-conditioning system of the swimming pool hall), as well as sanitary and hygienic procedures applicable to the given type of swimming pool.

The most important differences in the impact of the studied parameters on the content of THMs and CC were found in hot tubs (HT1 and HT2), the outdoor pool (ORP), and the scuba-diving pool (SDP). In the case of HT1 and HT2, the high water temperature and the high bather loads caused CC content to be the highest there. In addition, comparison of the total THM content in HT1 and HT2 pools with a similar function, but with a different capacity (2.4 m^3^ in the case of HT1 and 5.6 m^3^ in the case if HT2), showed significant difference in the total THM content. In HT1, it was more than twice as high as in HT2 (0.052 mg/L in HT1 and 0.026 mg/L in HT2).

In the case of ORP, the contact of water with air and the effect of wind caused THM and CC content to be relatively small. In the case of SDP, due to its unusual function and the ensuing small attendance, and hence the large water volume per person, THMs and CC were also small.

It should be emphasized that the results of research on changing swimming pool operating conditions may lead to surprising and often ambiguous conclusions.

The authors conclude that research on the pool water quality in the actual working conditions of swimming pool facilities, despite the fact that many factors need to be taken into account, is necessary due to the need to preserve the health safety of swimmers and, what is also extremely important, the staff.
